# A flexible approach to assess fluorescence decay functions in complex energy transfer systems

**DOI:** 10.1186/s13628-015-0020-z

**Published:** 2015-04-03

**Authors:** Christoph Roethlein, Markus S Miettinen, Zoya Ignatova

**Affiliations:** Biochemistry and Biology, University of Potsdam, Potsdam, Germany; Theoretical Physics, Free University Berlin, Berlin, Germany; Biochemistry and Molecular Biology, University of Hamburg, Hamburg, Germany

**Keywords:** Time resolved FRET, Monte-Carlo simulations, Complex heterogeneous systems, Protein aggregation

## Abstract

**Background:**

Time-correlated Förster resonance energy transfer (FRET) probes molecular distances with greater accuracy than intensity-based calculation of FRET efficiency and provides a powerful tool to study biomolecular structure and dynamics. Moreover, time-correlated photon count measurements bear additional information on the variety of donor surroundings allowing more detailed differentiation between distinct structural geometries which are typically inaccessible to general fitting solutions.

**Results:**

Here we develop a new approach based on Monte Carlo simulations of time-correlated FRET events to estimate the time-correlated single photon counts (TCSPC) histograms in complex systems. This simulation solution assesses the full statistics of time-correlated photon counts and distance distributions of fluorescently labeled biomolecules. The simulations are consistent with the theoretical predictions of the dye behavior in FRET systems with defined dye distances and measurements of randomly distributed dye solutions. We validate the simulation results using a highly heterogeneous aggregation system and explore the conditions to use this tool in complex systems.

**Conclusion:**

This approach is powerful in distinguishing distance distributions in a wide variety of experimental setups, thus providing a versatile tool to accurately distinguish between different structural assemblies in highly complex systems.

**Electronic supplementary material:**

The online version of this article (doi:10.1186/s13628-015-0020-z) contains supplementary material, which is available to authorized users.

## Background

FRET is a powerful approach that is widely used to assess structural dynamics in biomolecules. FRET is based on non-radiative energy transfer between two dyes dependent on their absolute distance [1,2] which provides specific information about distances between dye-labeled macromolecules and on structural characteristics of investigated systems [3-5]. For many years it has been used to differentiate a small set of defined states, e.g., in protein folding studies [6] and co-localization of biomolecules in cells [7]. The shape of distance-dependence distribution is increasingly exploited for more detailed analysis. For complex FRET systems, e.g. membranes or spherical vesicle surfaces, some analytical solutions have been developed to determine distance distributions for various shapes [8-10]. However, it has been increasingly recognized that more information can be extracted from time-resolved fluorescence based approaches [3,11]. In heterogeneous biological systems with complex distance distributions, monitoring FRET in a time-resolved manner provides ample information on the variety of donor surroundings and allows detailed differentiation between distinct structural geometries. Various approaches which differ in their complexity and utility have been developed to extract different parameters from time-correlated FRET measurements. Monte Carlo-based methods have been applied to achieve higher level quantification of FRET experiments; examples include accurate determination of distributions of membrane components and assessment of FRET efficiency in complex systems with multiple transfer opportunities in large actin-filaments [12,13]. The ExiFRET tool (www.exifret.com) developed by Corry and co-workers is capable of calculating FRET efficiencies and ratios between donor and acceptor emission in a large variety of FRET-based microscopy studies using the excluded circular volumes around the dye labels [14]. However, this tool is limited to only predicting the averaged FRET efficiency of a system.

Recent developments include Monte Carlo analysis of FRET kinetic traces which are used to estimate FRET rates taking into account the full statistics of individual photon absorption, energy transfer, and photon emission events [15], or to gain detailed insight into the dynamics of labeled proteins switching between two states [16]. The algorithms and tools developed in this context focus on the precise prediction of the behavior of single FRET pairs and exact distributions of distances and relative orientations. However, they require molecular dynamics simulations to precisely predict the distance distributions and thus are limited to simple FRET systems that are manageable with moderate computational resources.

Neurodegenerative pathologies based in misfolding and aggregation, such as Alzheimer's and Huntington's diseases, are growing in medical prevalence [17]. Aggregation is a multi-step, heterogeneous process [18,19] and poses challenges for structural and mechanistic insight. A set of methods utilizing distance-dependent FRET is suitable to assess aggregate structure. Yet, it is limited in application because of lack of uniform algorithms and tools to extract distances from FRET-based measurements in such heterogeneous systems. In a recent study, we developed Monte Carlo simulations to deconvolute complex fluorescent decay signatures observed in polyglutamine fibril architecture [20]. As this approach is based on first principles of underlying fluorescence phenomena, its utility is generalizable in distinguishing between distance distributions of fluorescently labeled entities in a wide variety of experimental setups. Here, we extended the broad application of this algorithm to other heterogeneous systems in an accessible and open source platform. This method is applicable for predicting the time-correlated fluorescent signal for systems with multiple donors and acceptors within close proximity. We assessed the generalized conditions and assumptions under which the tool can be utilized without compromising the simulation results. Furthermore, we carefully investigated the impact of various assumptions that reduced the required input and the number and complexity of control measurements. The algorithm is a versatile tool for assessment of complex structural assemblies and requires considering detailed competitive transfer reactions between multiple donors and acceptors in combination with other experimental models given by molecular dynamics simulations, x-ray diffraction patterns or NMR spectroscopy.

## Methods

### Choice of dyes

We first selected a set of dyes for optimal consistency of simulated and experimental donor TCSPC histograms and to reflect the expected behavior in the simulations. Note that this approach allows also for calculating the acceptor TCSPC histograms; here, if not otherwise mentioned, we focus primarily on the donor TCSPC histograms. Typically, the Förster radius of the FRET pair should be chosen within a range of distances which are expected between the donor and its nearest neighboring acceptor for the following reasons. Firstly, FRET efficiency and measured donor TCSPC histograms respond sensitively to distances ranging from one half to two times the Förster radius, thus increasing the influence of small deviations between distances. For distances beyond twice the Förster radius, the FRET rates are extremely low with very little influence on time-dependent photon emission probability. Energy transfer for distances closer than half of the Förster radius is extremely efficient, but hardly any donor photons will be collected. Typically, those distances do not influence the donor photon distributions, since donors with very high quenching rates have negligible brightness compared to donors without acceptors at distances closer than half of the Förster radius. Additionally, since within the half of the Förster radius, the donor-to-acceptor transfer occurs almost instantaneously, no significant change in the donor TCSPC (or even in the acceptor TCSPC) is expected. Secondly, the factor describing the relative orientation of donor emission and acceptor absorption transition dipoles (*κ*^2^) is usually taken as 2/3, which is based on the assumption that the dyes have enough time to randomize their orientation during the time the donor stays excited [1]. Shortening the fluorescent decay time by introducing acceptors in much closer proximity than the half of the Förster radius renders this assumption about *κ*^2^ inaccurate. Thirdly, too many short range distances dramatically increase the simulation time by reducing the number of the collected photons because of the large competition rates for transfer deactivation of the donors. Furthermore, the fluorescence decay time of the donor should be sufficiently long during the measurements to provide enough time for the donors to randomize their orientation after excitation and limit the fraction of photons detected by a non-random distribution of photon emission angles at the onset of signal detection.

The possibility of homo-FRET adds other aspect to consider when selecting the dyes. Usually, homo-FRET decreases the time needed for randomization of the orientation of the excited dyes and introduces randomness in the emission direction of transition dipole moment of the donor. Furthermore, homo-FRET changes the position of the excited donor which is usually fixed in the simulation. An appropriate choice of the dye will render this effect negligible. The Stokes shift should be sufficiently large to minimize the amount of homo-FRET in the fluorescent decay time measurements. The effects of transferring the excited states between two neighboring donors in either direction do not necessarily cancel out each other. Therefore, the overlap of the absorption and emission spectra of the donor should be minimal to limit the donor-donor transfers to distances close to zero in which both donors would almost have the same environment.

Here, we used Alexa 488 and Alexa 594 (Life technologies). The dyes were chosen because they implement the described requirements and criteria for suitable experimental setups, e.g. high photostability, extinction coefficient and quantum yield [21,22].

### Generation of input files

To read the information of the positions serving as an anchor point into the code, input files are created according to the following scheme. The files contain the three Cartesian coordinates that define the dimensions of the simulated volume, three binary values determining the periodicity of the system in each direction and the number and Cartesian coordinates of donors and acceptors in the system. All coordinates are given in nanometers.

We randomly positioned our dyes according to the probabilities derived from (i) specific dye concentrations with randomly distributed dyes in the three dimensional space, or (ii) fixed labeled structures with known labeling sites according to the probabilities of labeling with donors or acceptors. Additionally, files with defined donor-acceptor distances in defined ratios were created to compare the simulations to theoretically calculated fluorescent decay time behavior.

### Calculation of FRET rates

**Figure 1:**
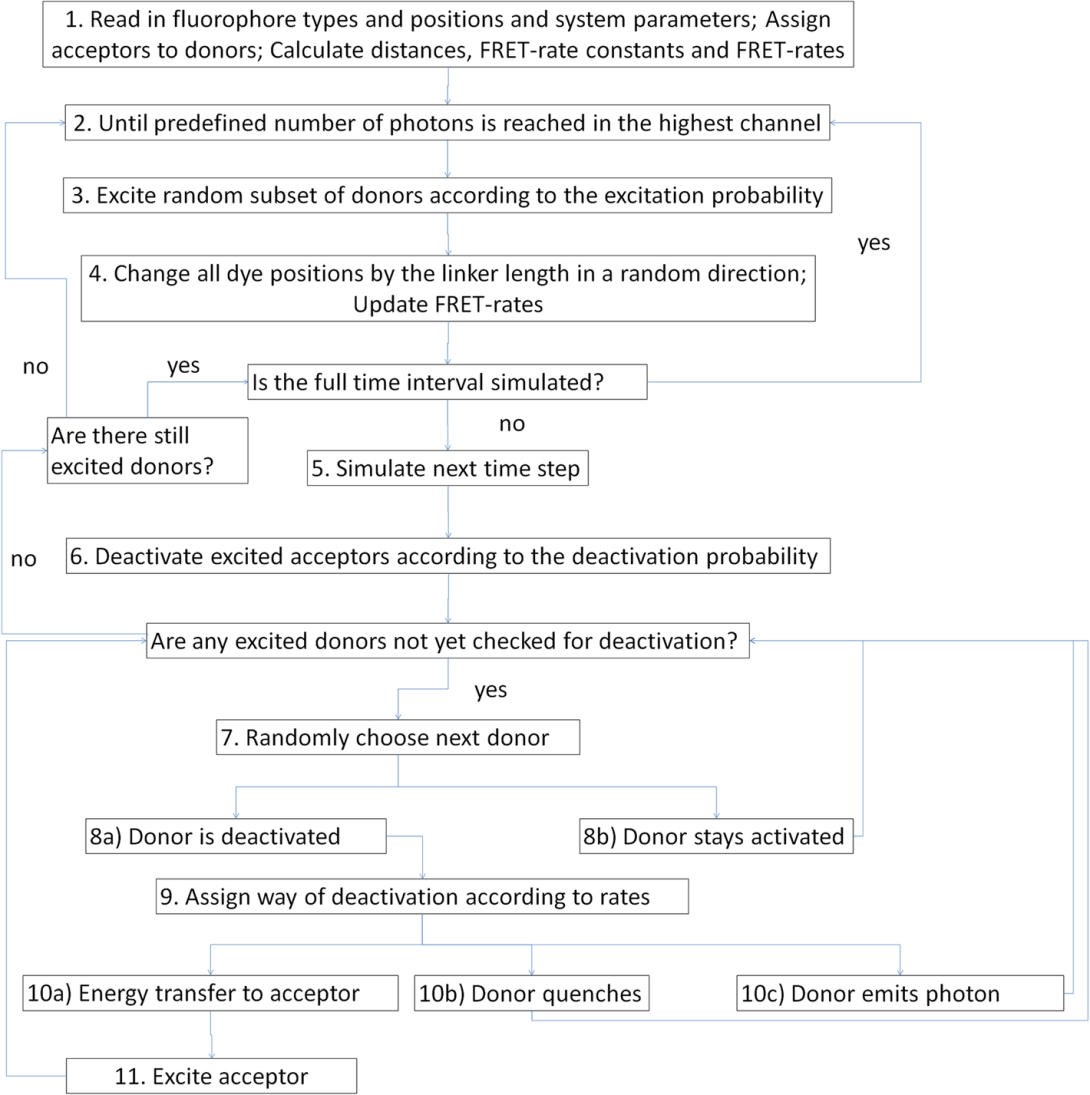
**Schematic flow chart depicting the simulation routine.**

By simulating of the possible deactivation processes for each donor, all acceptors that can get possibly closer than twice of the Förster radius, considering also the length of the dye linkers, are taken into account. The distances *r*, the FRET rates *k*_*T*_ and the deactivation probabilities *P*_deactivation_ are then calculated for every donor (Figure 1, Step 1).

1 $$ {k}_T=\frac{1}{\tau_{D,0}}\times {\left(\frac{R_0}{r}\right)}^6 $$

where *τ*_D,0_ is the donor lifetime in absence of acceptor and *R*_0_ is the characteristic Förster radius, at which half of the deactivation of electronically excited donor states occurs via energy transfer to the acceptor.

2 $$ {P}_{deactivation}=1- \exp \left(-{\displaystyle {\sum}_i{k}_i}\times \varDelta t\right) $$

where *k*_*i*_ denotes the individual deactivation rates for this particular donor by photon emission, FRET, or other deactivation processes and Δ*t* is the length of the time interval. The periodicity of the volume is taken into account to correctly determine the environment of every donor. [For additional information see Additional file 1.]

The linker distances can be chosen for the donor and acceptor individually. In the simulation, the position of the dyes is changed from the coordinates of the labeling site given in the input file by that distance in a random direction (Figure 1, Step 4). During a single excitation cycle the dye positions are assumed to be constant. However between two excitation events all dyes are moving within the volume which is limited by the linker independent of their last assigned position. Note that this leads to rather complex distance distributions between the dyes resulting in uneconomical computation times for analytical solutions. A short linker distance will minimize errors due to non-random orientations of the linker and simplifications of the distance distribution of the dye to its original coordinates. In contrast, larger linker sequences allow for larger inaccuracies in the predicted distribution of dye positions. However, a severe violation of the assumed conditions would be to restrict the randomness of the dye orientation or its rotational flexibility. This could be the case by a very short or very stiff linker sequence. Restricted dye rotation flexibility will result in false predictions of *κ*^2^ values and, at least during the early time steps, the time-dependent detection probability of the emitted photons at the chosen detection angle. We recommend ensuring reasonable dye flexibility by confirming the dye randomization by anisotropy measurements [20]. Alternatively, by restricted dye-dipol mobility accurate molecular dynamics (MD) simulations should be performed to obtain the exact distribution of the relative angles in each system individually [16]. Note that MD simulations might need an extraordinary computational power and thus might not be applicable in complex systems.

### Simulation routine

After every necessary parameter is read or calculated, the experimental procedure is simulated (Figure 1, Step 2-11). A random subset of donors is chosen by assigning random numbers to each donor and excited at a fixed time point zero, assuming an infinitely short light pulse for excitation (Figure 1, Step 3). To take into account the chronology of events, every time step observed in the experiment is simulated for all dyes (Figure 1, Step 6-11). After simulation for a time equal to the whole experimentally monitored time or when no excited donor has remained in the excitation cycle, the circuit is terminated. Optionally, the time dependence of the acceptor deactivation is also simulated over the whole time interval. The excitation cycles are repeated until reaching in one time interval the threshold of photons which is set by the user and are needed to collect for proper statistics (Figure 1, Step 2). Within each time step of a given excitation cycle the donors and acceptors are monitored in the given order (Figure 1).

### Acceptor deactivation

To allow acceptors that had already received an energy quantum to become available for new FRET events, we assign a random probability to every electronically excited acceptor using a random number generator (Figure 1, Step 6). If it is lower than the deactivation probability within a simulation time step, the acceptor is again available for energy transfers in analogy to Eq. 2 where the acceptor lifetime is the reciprocal sum of the deactivation processes. The number of deactivation events of acceptors is summed up for each time interval.

### Donor excitation cycles

To every donor in the system a random value is assigned with a random number generator. Donors with random values below the excitation probability are excited. We accelerated the simulation by ensuring that there is at least one donor excited in every excitation cycle. Excited donors are subsequently ordered by their randomly assigned value, from the lowest to the highest, and in a given excitation cycle simulated in that order to avoid a possible bias due to a fixed order in the simulated donors (Figure 1, Step 7). All acceptors within a distance twice of the Förster radius to a donor, considering the linker lengths of both donor and acceptor, are tested for their availability for FRET within this time interval. The probability to remain excited is then calculated for every donor based on its current environment (Eq. 2) and is compared with a random probability to remain excited or not (Figure 1, Step 8). In case of deactivation, the type of deactivation is determined by defining probability intervals from the ratio of the deactivation rates of different processes and by comparing to another random probability. The deactivation process representing that interval is chosen for deactivation (Figure 1, Step 9). The individual deactivation processes are summed up for each simulated time interval. It should be noted that all photons emitted during a single excitation cycle are counted for the given time interval. This is done because time-dependent variations of the photon detection probability (at the detection angle) are assumed to be of minor importance for the simulated systems; also in reality the frequency of photon counting should be low enough to not significantly contribute and detect more than one photon in an excitation cycle. If a donor is deactivated via a FRET event, the corresponding acceptor is excited and therefore temporally blocked for another energy transfer (Figure 1, Step 11).

### Output of the simulations

The output consists of all donor identities that do not have any acceptor in a relevant distance and includes also a table containing the frequencies of all donor deactivation processes observed in each time interval. The total number of acceptor deactivations is included in the output. The ratio of deactivation rates for each acceptor usually stays constant over time. Hence, the acceptor deactivation represents a qualitative TCSPC histogram, given the time-dependent excitation pattern of that FRET system. Note that the acceptor decays are only simulated completely if the excitation cycles are not terminated after deactivation of all donors. To compare the acceptor signal with experimentally recorded acceptor photon statistics and set them in relation to the observed donor signal, assuming a known ratio of detection probability by the two detection systems, only the quantum yield of the acceptor *Φ*_A_ needs to be determined.

3 $$ {\varPhi}_A=\frac{k_{Fluorescence}}{{\displaystyle \sum_i{k}_i}} $$

where *k*_Fluorescence_ depicts the rate of deactivation by photon emission. To compare with the experimental data, the simulated photon decay must be convoluted with the instrument response functions of the day of measurement for the given dye channel to implement the time dependence of the detection probability density of the instrumental setup.

### Fluorescence decay time measurements

Fluorescent decay times were recorded on a FL920 spectrometer (Edinburgh Instruments) operated in a TCSPC mode. In the measurements we used a time window of 50 ns and 1024 channels. Samples were excited at *λ*_ex_ = 450 nm using a SC-400-PP supercontinuum source (Fianum) and the emission was collected at *λ*_em_ = 525 nm using a polarizer set at magic angle position and a multichannel plate (Europhoton) as a detector. The repetition rate of the excitation light source was set to 10 MHz.

### Quantum yield and Förster radius determination

Using a photoluminescence (PL) quantum yield measurement system (C9920, Hamamatu Photonics), the quantum yield *Φ*_D_ for the donor was determined to be 0.6. Based on that, the Förster radius was determined to be 5.4 nm (Eq. 4) assuming that the randomization of the dye orientation is much faster than the fluorescence decay time. The refractive index was 1.33. The overlap integral *J* (*λ*) was calculated from the recorded donor emission and acceptor absorption spectra determined with the QuantaMaster 40 (Photon Technology International) with Felix32 software. The Förster radius is a function of *κ*^2^, describing the relative orientation of the donor and acceptor transition dipoles, the quantum yield of the donor *Φ*_D_, the refractive index *n* of the solution and the overlap integral *J* (*λ*) between the donor emission and the acceptor absorption spectra:

4 $$ {R}_0=\sqrt[6]{\frac{9\times \ln (10)\times {\kappa}^2\times {\varPhi}_D\times J\left(\lambda \right)}{128\times {\pi}^5\times {N}_A\times {n}^4}} $$

where *N*_A_ is the Avogadro number.

### Theoretical calculations

To verify the consistency of our code with the theory of Förster resonance energy transfer, we calculated the probability for photon emission. For multiple distances, the resulting distributions were set in relation to their weight according to the photons expected during the first time interval based on the probability to emit photons and on the fraction of excited donors in the particular surrounding.

5 $$ {N}_P(t)={\displaystyle {\sum}_i{f}_{D,i}}\times \frac{d{D}_{i, emission}}{dt}={\displaystyle {\sum}_i{f}_{D,i}}\times {k}_{emission}\times \exp \left(-{\displaystyle {\sum}_j{k}_j}\times \varDelta t\right) $$

where *N*_P_(*t*) is the amount of photons in dependence of time, *f*_D,i_ is the fraction of donors with surrounding *i*, *k*_emission_ is the emission rate of the donor and the sum of *k*j represents the combined deactivation rate constant for all deactivation processes. The derivative d*D*_i,emission_/d*t* represents the deactivations by emission per time unit at a given time point. It cannot be assumed to be constant due to photon decay events of donors with very close acceptors at earlier times than resolved by a typical experimental time resolution. Therefore in the first time interval it was determined for 1000 equal subintervals and subsequently averaged. [For additional information see Additional file 1.]

### Code access

The code is deposited in an open-access platform domain (http://figshare.com/) and has the accession number: 1158992 (http://figshare.com/articles/FRET/1158992).

## Results and discussion

Time-correlated photon count measurements offer a powerful tool to investigate dye surroundings. TCSPC histograms respond sensitively to environmental changes, such as solvent polarity, pH or presence of heavy atoms. Additionally, atomic distance information can be extracted using resonance energy transfer between donors and acceptors that are introduced at targeted labeling sites. However, when using a mathematical fitting function to gain many parameters it is often impossible to implement restrictions and the fitting will instead settle into different minima that represent meaningless dye arrangements. Connecting the knowledge of the possible distance distributions to fluorescent decay functions using Monte Carlo simulations provides a powerful way to bypass this limitation. It is also based on detailed mechanistic understanding of the underlying physical processes that can produce observed signals.

### Simulations predict the calculated fluorescent decays

**Figure 2:**
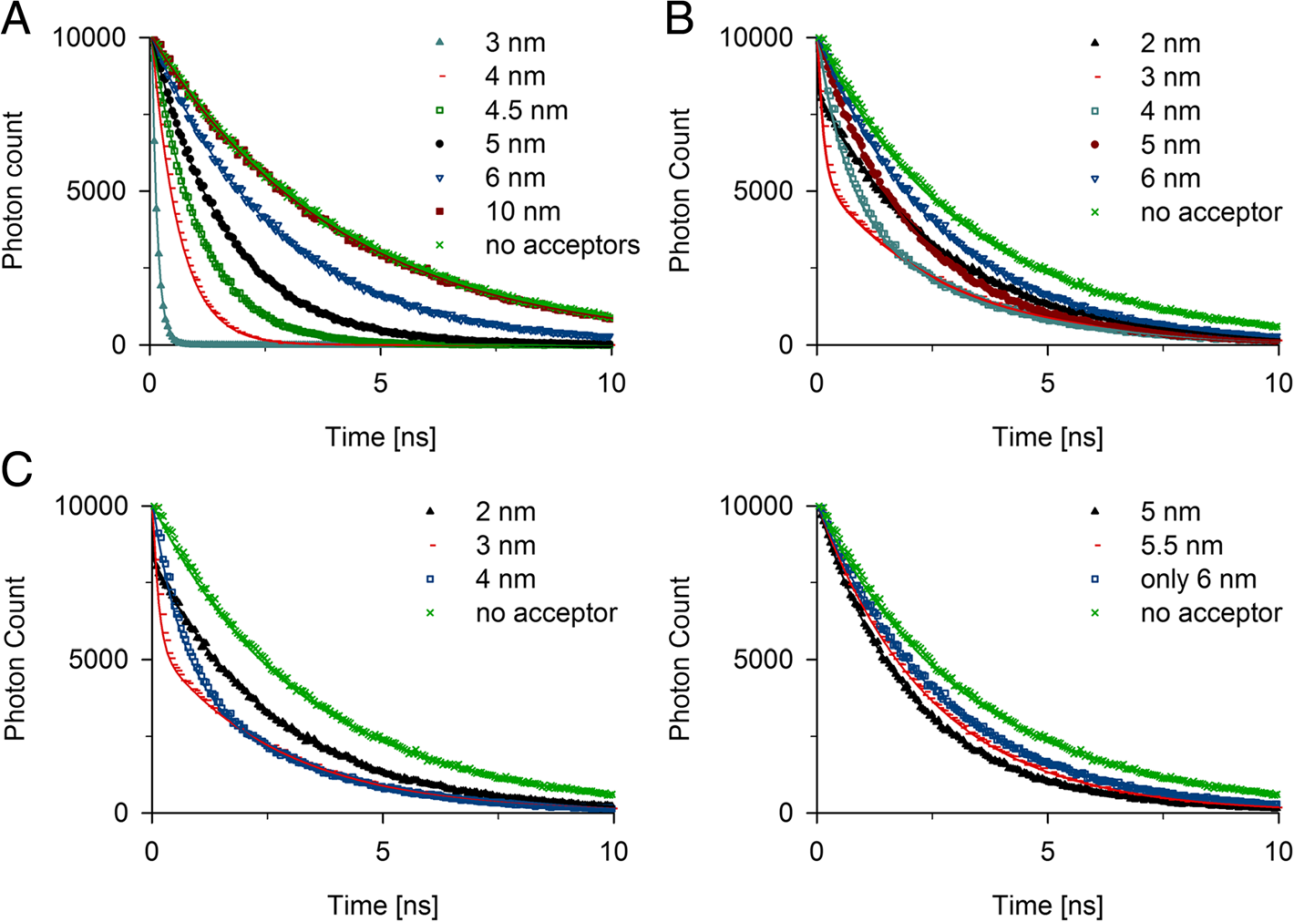
**Reproduction of theoretically calculated fluorescent decay times. A**: Simulation of time-dependent photon counts for single donor environments. The acceptor distances were between 1 and 10 nm. **B, C**: Simulation of fluorescent decay signals of equal amounts of FRET pairs (from A). Representative curves of simulations with one donor-acceptor distance fixed at 4 nm **(B)** or 6 nm **(C)** while for the other donor-acceptor pair the distance was variable and the distances are displayed in the legend. For better representation of the 6-nm experiment the curves are split in two plots. The amount of the two donors in each simulation was equal. No acceptor denotes a simulation experiment with donor only. Simulated values are depicted with symbols and the matching theoretical calculations as lines in the same color.

To evaluate the predictions made with our code, we first performed simulations with simple dye systems whose fluorescent decay function can be calculated (Eq. 5). We created input files with one (Figure 2A) or two (Figure 2B, and C) distinct donor environments. The acceptor was simulated for each donor environment at defined distances with 0.5 nm step (Figure 2). Notably, for a single donor environment all fluorescent decay functions were predicted accurately and precisely (Figure 2A).

For the two donor environments we simulated all possible combinations of pairs from the single donor environment to ensure that the code will weight them according to the expected stochasticity (Figure 2B and C). Note, that even for fluorescent decays, where one of the distances was changed by 0.5 nm, the predictions were clearly distinct (Figure 2B and C). As long as the distances dwelled between half and twice the Förster radius, the predicted photon distributions mirrored the calculated TCSPC histograms. However, if one of the distances was close to those boundaries, a slight change in the distance resulted only in minor differences in the theoretically expected curves (for example Figure 2A, 10 nm and no acceptor); either the whole signal (for short distances) or almost none of it (for large distances) was quenched.

A fraction of donors with acceptors at a distance of 2 nm or less had very little influence since the amount of photons contributed by such donors was small. Therefore, the calculated decay functions were mainly influenced by other donor environments. That led to a paradoxical situation: a slight increase of those donor-acceptor distances beyond 2 nm, which resulted in less quenching, seemed to increase the quenching effect by enhancing the relative contribution to the total signal (Figure 2B and C). However, increasing the dye distances led to less quenching of that donor fraction, which resulted in longer fluorescent decay times (Figure 2C).

### Random dye distribution in the 3D-space

**Figure 3:**
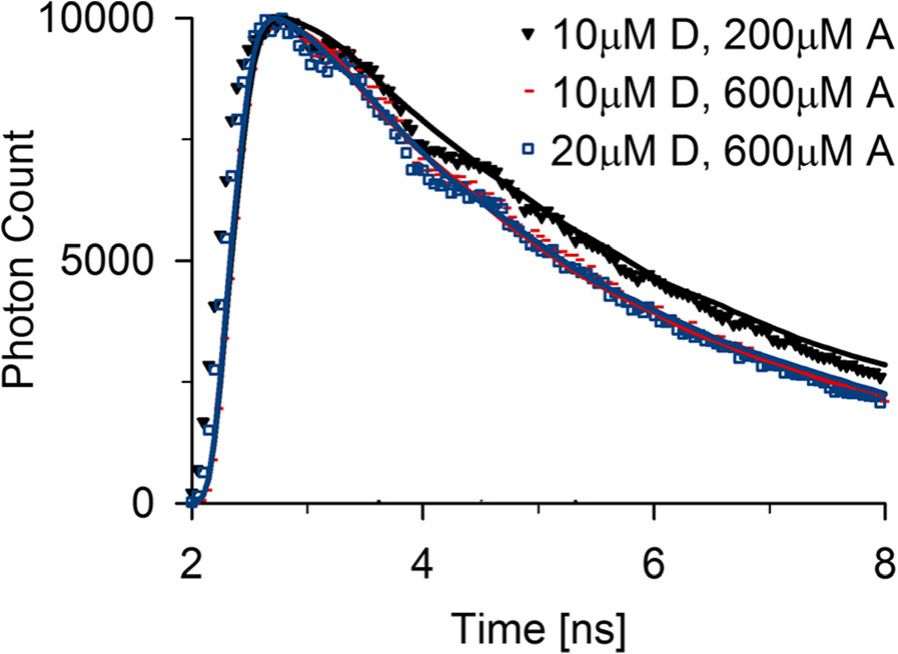
**Reproduction of the experimental fluorescent decay times.** Fluorescent decay times of random 3D-distributions were measured by mixing fluorescent dyes in solution, and were separately predicted with our Monte Carlo simulations. Note that if the experimental fluorescent decay reaches the noise level, the simulations will approach zero value, thus only experimental data points above the noise level should be considered. The experimental values are depicted with symbols and the matching calculations as lines in the same color.

Next, we created a system in which the dye distances follow a distribution instead of being fixed to discrete values. We therefore used a simple experimental system containing Alexa488- and Alexa594-maleimides with defined concentrations. High dye concentrations were used in order to observe significant quenching. As expected, increasing the acceptor concentration shortened the fluorescent decay times. An increase of the concentration of the donor, however, did not have any effect, since the donor excitation rates were chosen to be very low. The presence of additional donors did not change the donor environment as long as the excitation was sufficiently low to avoid competition for nearby acceptors (Figure 3). The TCSPC histograms were predicted by simulating a hypothetical dye solution with the equal amounts of donor and acceptor per volume unit compared to the experiments which were randomly distributed throughout the simulated volume.

Transfer processes between the dyes outside FRET are not considered in our code. The parameters and fluorescent dye pair should be chosen accordingly to minimize the influence of processes such as homo-FRET or Dexter energy transfer. When the expected dye distances are in the range of the Förster radius and the donor or acceptor properties are suitably chosen, ignoring these processes should not influence the simulation results.

### Influence of the linker length

**Figure 4:**
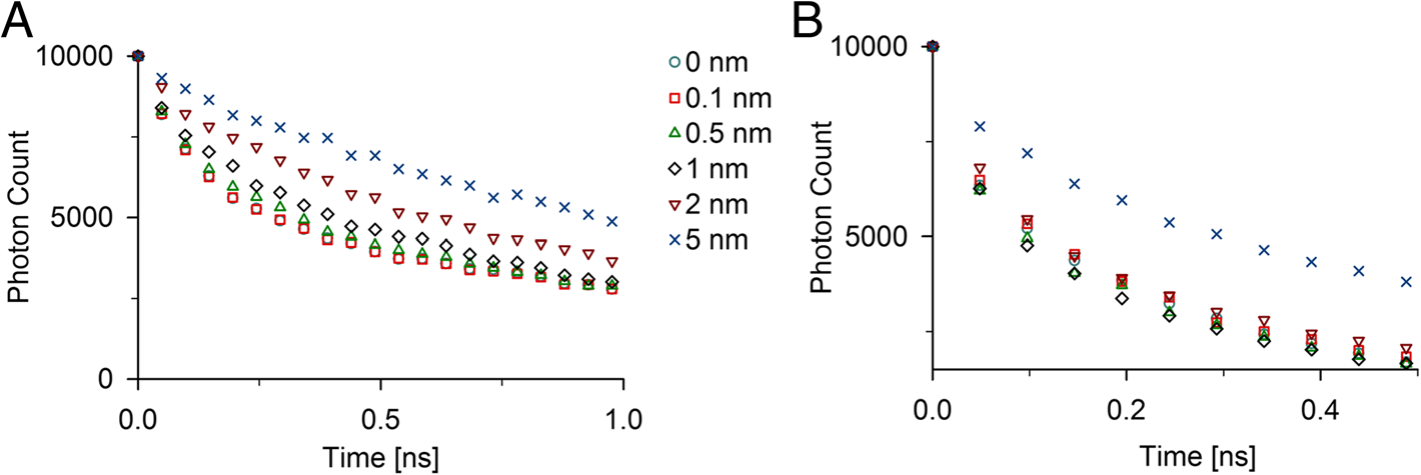
**Influence of the linker length.** Changes in TCSPC histograms were investigated as a function of different lengths of linker between dyes and labeling positions. These differences were based on equal amounts of FRET pairs with either 3 nm or 6 nm distance between the labeling positions **(A)** or distance positions of a polyglutamine fibril model **(B)** as described in [20]. Note that the time scales are different in order to better resolve an important area of signals before reaching the background.

Subsequently, we assessed the relevance of predicting the correct change in distances for dyes attached to fixed positions (Figure 4). Since the dyes should rotate freely, it is crucial to allow for some freedom in rotation and attach them via a linker sequence. Hence, it is also crucial to run the simulation by allowing for similar freedom in the dye coordinates. We tested the impact of changing dye positions on single FRET pairs and on a model structure of amyloid polyglutamine fibrils with multiple FRET interactions [20].

The dye positions were reassigned to a sphere around the labeling position with different radii. Allowing for changes in the dye positions altered the shape of the fluorescent decay function (Figure 4). For single donor-acceptor pairs, the average distance increased relative to fixed positions, and further increased with the linker length (Figure 4A). Interestingly, changes in the dye positions became significant only when they reached values closer to the Förster radius. For the simulated Förster radius in the fibril environment of 4.7 nm, a significant influence was only observed for changes in dye distances above 1 nm. Convoluting the signal with the instrument response function would make those changes even less significant.

For complex FRET systems, e.g. amyloid fibrils [20], where the acceptors move in any direction (towards and away from donors), the influence was somewhat different (Figure 4B). Small isotropic changes in position increased the quenching behavior, most likely due to the shorter distances than the distance between labeling sites. The effects of larger and smaller distances do not cancel each other out and instead result in higher donor quenching which is determined by the shape of the distance dependence of FRET efficiency.

Additionally, FRET pairs labeled at positions closer than the half of the Förster radius had higher contribution to the signal. If some dyes change their position and influence the donor-acceptor distance growth, it will result in a relative increase in the number of photons detected shortly after excitation. For larger linker length, however, the average distance was dominated by another effect. The total volume accessible to the dyes increased in non-periodic systems in all three dimensions, thus reducing the effective dye concentration and increasing the average dye distances. At some point, the original distances between the dyes will become insignificant, since they are comparably small to the length of the flexible linker. This is a limitation that needs to be carefully considered when designing a system to be assessed with this approach.

In sum, allowing for changes in the dyes positions can influence and potentially interfere with the success of the simulations, albeit within a limited distance scale. However, by linker size small compared to the Förster radius, it had minor influence on complex dye systems with high label densities. Therefore, more precise assumptions, such as effective ranges of linker-length rather than fixed length defined by theoretical models or molecular dynamics simulations, or excluded dye volumes based on angle restriction from the volume occupancy, are not necessary for most applications.

### Photon detection limit

**Figure 5:**
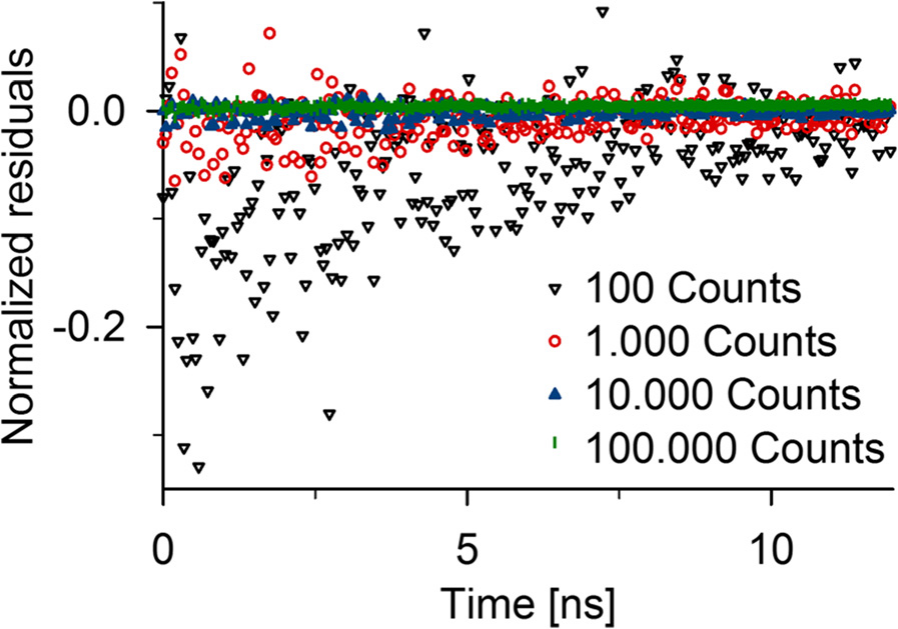
**Influence of collected photon number on the stochastic accuracy.** Residuals of simulations based on 100; 1000, 10000 or 100000 photons collected in the most frequently detected time interval.

To investigate how much signal needs to be obtained for stochastically reliable sample size, we performed a simulation using only Alexa 488 labeled protein within our polyglutamine amyloid fibrils [20] and investigated the effect of the number of collected photons. Plotting the residuals in relation to the expected values as a function of time revealed an unsatisfactory result in the early time intervals for the low photon statistics (Figure 5). Compared with the theoretical fluorescent decay times, the photon counts for single time intervals deviated by up to 30% when small amounts of photons were collected. In addition, for those early time intervals the photon counts seemed to be systematically underestimated. An outlier that collects many photons will terminate the simulation prematurely. Thus, collecting substantially more photons decreased the impact of those outliers and the simulations run sufficiently long. Thereby, deviations from the theoretical predictions were randomly distributed around the expected values which collected more reliable amounts of photons. The residuals also decreased with higher photon counts. Importantly, the bias of underestimating the expected number of photons vanished when collecting at least 10000 photons.

### Excitement probability

**Figure 6:**
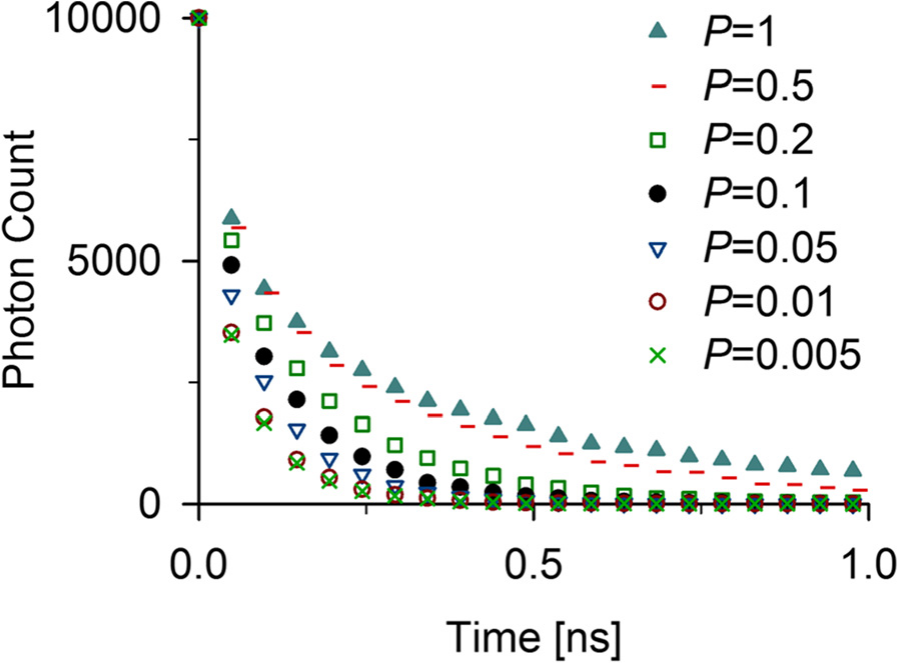
**Influence of excitation probability on the simulations.** Simulations are based on excitement probabilities between 0.5% and 100%.

In time correlated single-photon count measurements, the probability *P* to obtain a photon within an excitation cycle is typically very low. This is necessary because in a given excitation cycle only one photon can be detected. If more photons would reach the detector within one excitation cycle, only the first photon would be counted, leading to a bias and thus to a misrepresentation of the time-dependent photon distribution. The probability to detect more than one photon should be negligibly small. In our Monte Carlo simulation we can simultaneously monitor several photons in the same excitation cycle. Given that the typical small fraction of excited donors in the experimental setup slows down the simulation, it is reasonable to increase the probability for donor excitation artificially, although the correct value can be easily calculated using:

6 $$ {P}_{excitation}=\frac{I\times \varDelta t\times A}{E_P\times {N}_D}\times \left(1-{10}^{\frac{\varepsilon_D\times l\times {N}_D}{V}}\right) $$

with *I* – irradiance, *Δt* – the time interval of the excitation light pulse, *A* – the surface area of the light pulse, *E*_P_ – the energy of a single photon, *N*_D_ – the number of donors in the simulated volume *V*, *ε*_D_ – the extinction coefficient of the donor, and *l* – the length of the hypothetically excited volume in direction of the light beam. Despite the capability of detecting multiple photons without any bias, the simulations will be affected by increasing fractions of excited donors. An increase of the amount of excited donors also results in an increased amount of electronically excited acceptors during an excitation cycle. In situations where two donors in close proximity competing for the same acceptor are too frequent, they could induce error in the prediction of the donor TCSPC histograms. Thus, we addressed at which excitation probability such competition would alter the time-dependent profile of the photon emission. For this purpose, we simulated the structure of polyglutamine fibrils composed of 20% of donor-labeled monomers and 20% of acceptor-labeled monomers. The simulated fluorescent decay function was dramatically influenced with increasing excitation probabilities (Figure 6). Changing the excitation probability to 1%, which is orders of magnitude above a typical experimental excitation probability, had a minor influence on the fluorescent decay. It should be noted that this boundary can slightly shift at different dye concentrations and for a new system repeating the test should be considered. Moreover, moving the excitation probability to a maximum that does not alter the fluorescent decay time is advisable, since it results in substantially shorter simulation times.

## Conclusions

In conclusion, our approach is a step forward in exploiting the power of FRET to determine distances at the molecular level. The Monte Carlo-based simulation tool that we developed to predict the time-correlated single photon counts correctly assesses the donor behavior and allows for evaluation of precise structural models in complex FRET systems. Different key input parameters can change the predictions and reliability of the simulations. For a stochastically meaningful simulation at least 10,000 photons should be collected in the most frequently observed time interval. The simulation time can be shortened by artificially increasing the excitation probability of the donors in the simulated system. For bulky dye systems whose dimensions largely exceed the relevant distance for FRET, including a flexible linker flattens the distance distribution and alters the resulting photon decay. For isolated donor-acceptor pairs, increasing the linker length systematically reduces the quenching as previously observed [16]. Taking into account the above mentioned restrictions and appropriate choices of parameter this tool bears a broad utility to validate structural models for a variety of complex system. Importantly, this tool is also capable of studying dye systems with dimensions not suitable for detailed molecular dynamics simulations.

### Ethics statement

This research does not involve human subjects, human material, or human data.

## Additional file

Additional file 1:
**Additional information on the derivations of equations** 2 **and** 5 **.**

## Abbreviations

FRET: Förster resonance energy transfer
TCSPC: Time-correlated single photon counts
MD: Molecular dynamics
