# Supplementary material for: A flexible approach to assess fluorescence decay functions in complex energy transfer systems
Source: BMC Biophys. 2015 Apr 3;8:5. doi: 10.1186/s13628-015-0020-z (PMC4403788; doi:10.1186/s13628-015-0020-z)
Supplement: Additional file 1: — Additional information on the derivations of equations 2 and 5 . [file 13628_2015_20_MOESM1_ESM.pdf]

Additional information on the derivations of equations 2 and 5

$$\text{Donor}_{\text{excited}} \xrightarrow{k_{\text{radionless deactivation}}} \text{Donor}_{\text{deactivated}}$$

$$\text{Donor}_{\text{excited}} \xrightarrow{k_{\text{emission}}} \text{Donor}_{\text{deactivated}} + \text{Photon}$$

$$\text{Donor}_{\text{excited}} + \text{Acceptor}_{\text{deactivated}} \xrightarrow{k_{\text{FRET}}} \text{Donor}_{\text{deactivated}} + \text{Acceptor}_{\text{excited}}$$

$$\frac{d[Donor_{excited}]}{dt} = -(k_{deactivation,radiationless} + k_{emission} + k_{FRET}) \times [Donor_{excited}]$$

$$[Donor_{excited}](t) = [Donor_{excited}]_0 \times \exp^{-(k_{deactivation,radiationless}+k_{emission}+k_{FRET})t}$$

$$\frac{d[Donor_{excited}]}{dt}(emission) = k_{emission} \times \exp^{-(k_{deactivation,radiationless}+k_{emission}+k_{FRET})t}$$

$$\frac{d[Donor_{excited}]}{dt}(FRET) = k_{FRET} \times \exp^{-(k_{deactivation,radiationless}+k_{emission}+k_{FRET})t}$$

$$\frac{d[Donor_{excited}]}{dt}(deactivation, radiationless) = k_{deactivation,radiationless} \times \exp^{-(k_{deactivation,radiationless}+k_{emission}+k_{FRET})t}$$
